# Serous Borderline Tumor Originating From a Paratubal Cyst in a 22-Year-Old Woman: A Case Report and Review of the Literature

**DOI:** 10.7759/cureus.116669

**Published:** 2026-09-21

**Authors:** Yan Li, Andrew Green, Rusella Mirza

**Affiliations:** 1 Pathology, Louisiana State University Health Sciences Center Shreveport, Shreveport, USA; 2 Obstetrics and Gynecology, Louisiana State University Health Sciences Center Shreveport, Shreveport, USA

**Keywords:** adnexal mass, braf mutation, paratubal cyst, salpingectomy, serous borderline tumor

## Abstract

Serous borderline tumors (SBTs) typically arise from the ovary, and their development within a paratubal cyst is an uncommon occurrence. We report a 22-year-old nulliparous woman with chronic dysmenorrhea and an enlarging right adnexal cyst who underwent laparoscopic bilateral salpingectomy for symptom relief and sterilization. Intraoperatively, a 5 cm right paratubal cyst was identified, with normal uterus and ovaries. Histopathologic examination revealed an SBT confined to the paratubal cyst (International Federation of Gynecology and Obstetrics stage IA (FIGO IA)). This case highlights that even small, radiologically benign-appearing paratubal cysts can harbor borderline neoplasia. Awareness of this rare entity and careful histopathologic evaluation of all adnexal cysts are essential for accurate diagnosis and optimal management in young women.

## Introduction

Paratubal cysts are cystic adnexal lesions that arise from the mesosalpinx, the upper portion of the broad ligament adjacent to the fallopian tube, accounting for approximately 5-10% of all adnexal masses [1-5]. They are typically small, thin-walled, and discovered incidentally during pelvic imaging or surgery [6]. Although sometimes grouped with paraovarian cysts due to their close anatomic proximity, paratubal cysts are distinct in origin, developing near the fimbrial end of the fallopian tube rather than adjacent to the ovary [7].

Most paratubal cysts are benign, but borderline and malignant transformations have been reported only rarely [3,8,9]. Serous borderline tumors (SBTs) are characterized by epithelial proliferation and nuclear atypia in the absence of stromal invasion [10]. While borderline ovarian tumors are relatively common, paratubal serous borderline tumors are exceedingly rare, with only a small number of cases reported in the literature, spanning a wide age range from adolescence to postmenopause and occurring predominantly in reproductive-age women [2,3,8,9,11].

We report one of the youngest adult cases of an SBT arising within a paratubal cyst, measuring less than 5 cm, incidentally identified during laparoscopic bilateral salpingectomy for dysmenorrhea and sterilization. This case underscores the importance of considering borderline pathology even in small, benign-appearing paratubal cysts and reinforces the value of thorough histopathologic examination of all adnexal lesions.

## Case presentation

A 22-year-old nulliparous woman (BMI 29.7) with a past medical history of anxiety and depression presented with longstanding dysmenorrhea and a progressively enlarging right adnexal cyst. Per the CHRISTUS Health CT chest/abdomen report from February 2025, an ovarian cyst measuring 2 cm, identified one year earlier, had increased to 6 cm. She reported persistent pelvic pain despite trials of combined oral contraceptive pills, which were discontinued due to intolerable side effects. The patient declined intrauterine device placement and expressed a strong desire for definitive surgical management due to ongoing symptoms and significant anxiety regarding the cyst. After counseling, she elected to undergo laparoscopic bilateral salpingectomy for management of presumed endometriosis and permanent sterilization. She and her husband, who had previously undergone vasectomy, had mutually decided against future childbearing.

Menarche occurred at age 14; however, her menstrual cycles had always been irregular, typically occurring every four to six weeks, with occasional intervals of up to three months. Family history was notable for breast cancer in her maternal grandmother at age 40. 

During laparoscopic bilateral salpingectomy, a 5 cm right paratubal cyst was identified adjacent to the right fallopian tube. The uterus, bilateral ovaries, and visualized bowel appeared grossly unremarkable. The specimen was submitted for pathologic evaluation and consisted of a 4.9 × 3.6 × 1.2 cm cystic mass with papillary excrescences adherent to the inner wall. The right fallopian tube was dilated and focally disrupted, while the left fallopian tube was grossly unremarkable.

Microscopic examination revealed an SBT arising within a paratubal cyst, with the adjacent right fallopian tube showing no significant pathology. The left fallopian tube was histologically unremarkable. The final diagnosis was SBT arising in a paratubal cyst, International Federation of Gynecology and Obstetrics stage IA (FIGO IA) (Figure 1).

**Figure 1:**
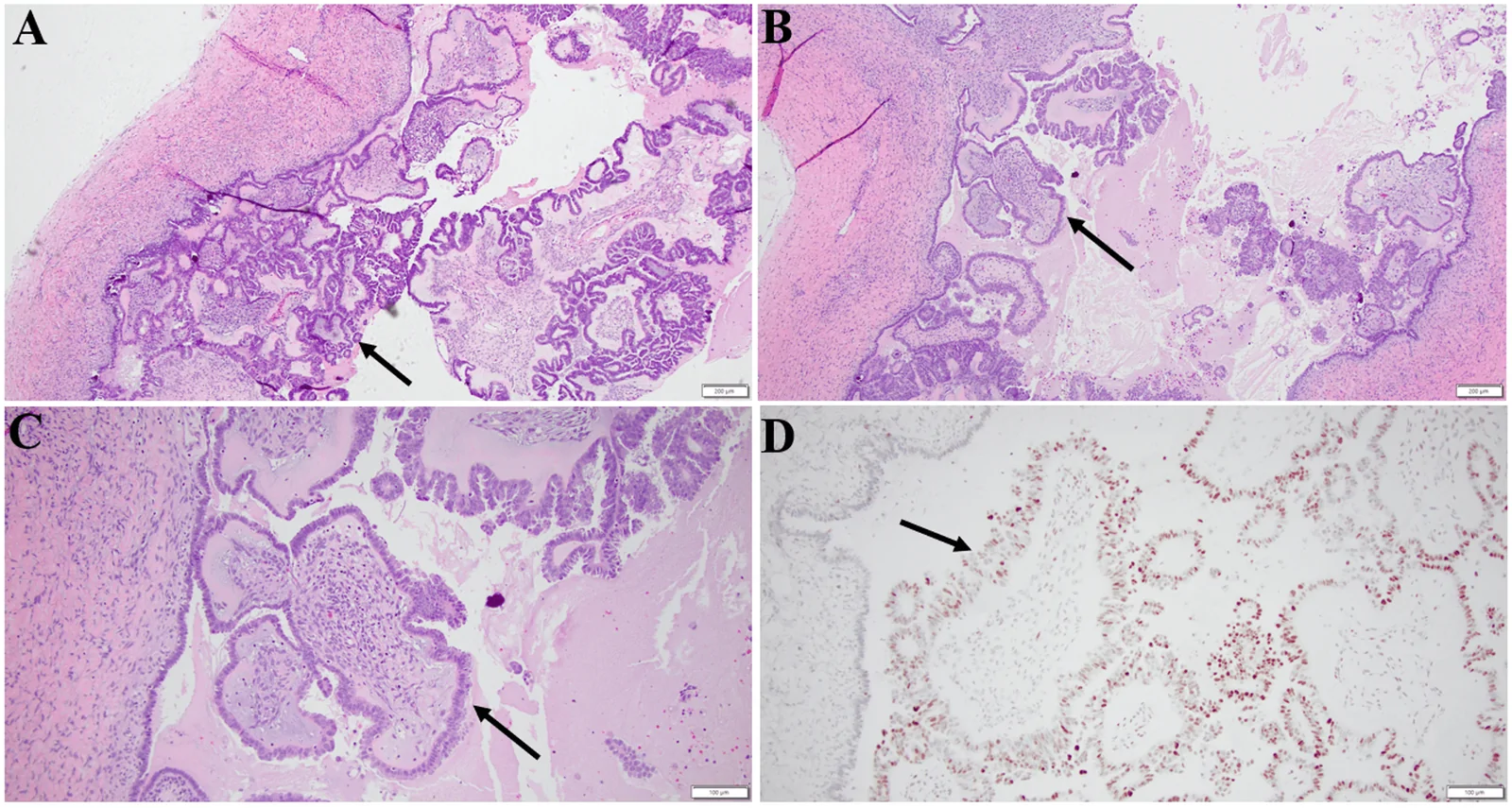
Histopathologic features of the paratubal serous borderline tumor (A) The cyst wall is lined by broad branching papillae with focal epithelial tufting (black solid arrow) and absence of stromal invasion (H&E, ×40). (B) Cyst papillary architecture with epithelial proliferation (black solid arrow) but preserved stromal integrity (H&E, ×40). (C) Proliferating serous epithelium shows stratification and tufting (black solid arrow) with minimal cytologic atypia and no stromal invasion (H&E, ×100). (D) p53 immunohistochemistry shows a wild-type (scattered) nuclear staining pattern (black solid arrow) (immunohistochemistry (IHC), ×100).

## Discussion

PSBTs are exceedingly rare lesions, with fewer than 15 well-documented cases reported to date [2-4,8,9,11-15]. Lee et al. reviewed eight earlier cases, including six serous, one endometrioid, and one mucinous borderline tumor arising within paratubal cysts [9]. Subsequent reports by Mehawej et al., Shin et al., Alaoui et al., Limaiem et al., and Chao et al. have further expanded the clinical spectrum [11-15]. Reported patients now range in age from 14 to 85 years, and tumor sizes range from 1 to 20 cm. Most presented with pelvic pain, torsion, or an incidental adnexal mass and were treated surgically without adjuvant therapy. Nearly all PSBTs have been confined to the cyst and staged as FIGO IA, although two were classified as FIGO IC due to intraoperative rupture [2-4,8,9,11-15]. Importantly, no recurrences or metastases have been reported during the reported follow-up periods, which extended up to 30 months, underscoring their indolent clinical course [2,3,8,11,13,15].

Our patient represents one of the youngest adults described and had a small (4.9 cm) cyst presumed benign preoperatively, which was subsequently found to harbor borderline histology following laparoscopic bilateral salpingectomy performed for dysmenorrhea and sterilization. Both ovaries and the uterus appeared grossly unremarkable, confirming a true paratubal origin. This case emphasizes that even small, clinically benign-appearing adnexal cysts can contain borderline epithelium, underscoring the importance of meticulous histopathologic evaluation of all excised adnexal lesions.

Preoperative distinction between paratubal and paraovarian cysts remains challenging, as imaging rarely delineates their precise anatomic origin. Sonographic features such as internal papillary projections or mural nodularity should raise suspicion for borderline or malignant transformation and warrant intraoperative frozen section evaluation, as emphasized by Lee et al. [9].

Histologically, PSBTs resemble their ovarian counterparts, showing hierarchical branching papillae lined by stratified serous epithelium with mild to moderate nuclear atypia and no stromal invasion [2,9,11]. In addition to the serous subtype, endometrioid and mucinous borderline tumors have also been reported in paratubal cysts [16,17], supporting the potential for diverse Müllerian differentiation within the mesosalpinx.

Molecularly, Chao et al. reported a PSBT in a 14-year-old adolescent, identifying a BRAF p.V600E mutation, suggesting that MAPK-pathway activation, a hallmark of ovarian SBTs, may also drive tumorigenesis in paratubal lesions [15]. Limaiem et al. described another case with morphologic parallels to low-grade serous ovarian tumors, reinforcing a shared Müllerian lineage [14]. To date, no chromosomal instability or germline alterations have been reported, consistent with their indolent clinical course [11,15].

Management of PSBTs remains primarily surgical. Most cases have been treated by complete excision, such as cystectomy or salpingectomy, without adjuvant chemotherapy or radiotherapy [2,11,14,15]. Minimally invasive approaches, including laparoscopy and laparo-endoscopic single-site surgery (LESS), have been successfully utilized and are considered safe and effective, offering reduced morbidity and excellent cosmetic outcomes [9]. Pelvic lymph-node sampling is generally unnecessary, as nodal metastasis has never been documented. Conservative, fertility-preserving surgery is appropriate for younger patients, provided that complete excision and staging confirm confinement to the cyst. Although recurrences have not been observed, several authors recommend long-term follow-up - up to 10 years - using periodic imaging and serum tumor markers such as CA-125 and CA 19-9 to ensure early detection of potential relapse [9,13,17].

In summary, this case expands the clinicopathologic spectrum of PSBTs by demonstrating that these tumors can occur in very young adults and even in small (<5 cm) cysts that appear benign radiologically. Our findings highlight that PSBTs represent a distinct entity within the continuum of Müllerian serous neoplasia and underscore the importance of routine histopathologic examination of all resected paratubal cysts.

A summary of previously reported paratubal borderline tumors, including the present case, is provided in Table 1.

**Table 1:** Summary of reported cases of paratubal borderline tumors with clinicopathological correlation FIGO, International Federation of Gynecology and Obstetrics; LESS, laparo-endoscopic single-site surgery

Author	Year	Age	Presentation	Procedure	Gross Finding	Pathology	Stage (FIGO)	Follow-up
Salamon et al. [16]	2005	45	Right adnexal mass	Laparoscopic bilateral ovarian cystectomy and partial right salpingectomy	3 cm twisted paratubal cyst	Endometrioid borderline tumor	IA	1 year, no recurrence
Seamon et al. [2]	2009	26	Pelvic pain, pelvic mass, torsion	Right salpingo-oophorectomy, omentectomy, appendectomy, bilateral pelvic-aortic lymphadenectomy	12 cm paratubal cyst	Serous borderline tumor	IC	1 year, no recurrence
Kumbak et al. [8]	2010	39	Left adnexal mass during C-section	Cystectomy, omentectomy, appendectomy, pelvic-paraaortic lymphadenectomy	6 cm paratubal cyst	Serous borderline tumor	IC	15 months, no recurrence
Shin et al. [12]	2011	27	Flank pain, right adnexal mass	Cystectomy, fertility-sparing comprehensive surgical staging procedures	16 cm paratubal cyst	Serous borderline tumor	IA	20 months, no recurrence
Terek et al. [3]	2011	19	Pelvic pain, torsion	Cystectomy, peritoneal washing	10 cm twisted left paratubal cyst	Serous borderline tumor	IA	7 months, no recurrence
Kiseli et al. [4]	2012	17	Complex right adnexal mass	Cystectomy and ovarian wedge resection	7 cm right adnexal mass	Serous borderline tumor	Not reported	1 year, no recurrence
Alaoui et al. [13]	2012	38	Pelvic pain, torsion	Cystectomy, hysterectomy, bilateral salpingo-oophorectomy, omentectomy	10 cm twisted paratubal cyst	Serous borderline tumor	IA	1 year, no recurrence
Lee et al. [9]	2016	17	Huge right adnexal mass	LESS salpingectomy and ovarian wedge resection	19 cm paratubal cyst	Serous borderline tumor	IA	3 months, no recurrence
Im et al. [17]	2011	20	Complex right adnexal mass	Laparoscopic paratubal cyst enucleation	10 cm paratubal cyst	Mucinous borderline tumor	IA	30 months, no recurrence
Mehawej et al. [11]	2020	85	Postmenopausal pelvic mass and bleeding	Total abdominal hysterectomy; bilateral salpingo-oophorectomy	1 cm paratubal cyst	Serous borderline tumor	IA	20 months, no recurrence
Chao et al. [15]	2021	14	Pelvic mass in adolescent	Cystectomy	20 cm right adnexal cystic mass	Serous borderline tumor (BRAF V600E+)	IA	3-month interval
Limaiem et al. [14]	2023	41	Pelvic mass	Cystectomy	9 cm paratubal cyst	Serous borderline tumor	IA	3-month intervals
Li et al. (present case)	2025	22	Long-standing dysmenorrhea; right adnexal cyst (5 cm)	Laparoscopic bilateral salpingectomy	5 cm right paratubal cyst	Serous borderline tumor	IA	Follow-up planned at 3-month intervals

## Conclusions

This case underscores that SBTs can arise within small, radiologically unremarkable paratubal cysts, even in very young women, and that neither patient age nor lesion size can reliably exclude borderline pathology. Because paratubal cysts are often regarded as clinically insignificant, this diagnosis can easily be overlooked without routine histopathologic evaluation of all excised adnexal tissue. Surgeons and pathologists should maintain a high index of suspicion for borderline neoplasia whenever papillary or nodular features are identified within a paratubal cyst, whether on gross inspection or frozen section. Given the favorable outcomes reported across the literature to date, complete surgical excision remains adequate treatment, though long-term clinical follow-up is prudent to confirm the durability of this outcome.
